# Sarcopenia screening in elderly with Alzheimer’s disease: performances of the SARC-F-3 and MSRA-5 questionnaires

**DOI:** 10.1186/s12877-022-03441-5

**Published:** 2022-09-17

**Authors:** Giulia Bramato, Roberta Barone, Maria Rosaria Barulli, Chiara Zecca, Rosanna Tortelli, Marco Filardi, Giancarlo Logroscino

**Affiliations:** 1 Center for Neurodegenerative Diseases and the Aging Brain, University of Bari Aldo Moro at Pia Fondazione “Card. G. Panico”, Tricase, Italy; 2 Department of Geriatrics, Fondazione Policlinico Universitario A. Gemelli IRCCS, Università Cattolica Sacro Cuore, Rome, Italy; 3 Department of Basic Medicine, Neuroscience and Sense Organs, University of Bari Aldo Moro, Bari, Italy

**Keywords:** Sarcopenia, Screening, Elderly, SARC-F-3, MSRA-5, Alzheimer’s disease

## Abstract

**Background:**

The 3-item SARC-F (SARC-F-3) and the 5-item Mini Sarcopenia Risk Assessment (MSRA-5) questionnaires have been recently proposed to screen elderly people regarding the risk of sarcopenia. However, no studies have investigated their performances in Alzheimer’s disease (AD).

**Methods:**

We conducted a single-center observational study, including 130 consecutive AD patients (mean age: 70.71 ± 8.50 y, 54.6% women) who attended a center for neurodegenerative diseases. Sarcopenia was diagnosed using the European Working Group on Sarcopenia in Older People of 2010 (EWGSOP1) and of 2018 (EWGSOP2) criteria. Sensitivity, specificity, positive and negative likelihood ratio, and the area under the receiver operating characteristic curve (AUC) were used to assess the diagnostic performance of SARC-F-3 and MSRA-5.

**Results:**

SARC-F-3 showed a sensitivity of 9.7%, a specificity of 82.8% and an AUC of 0.41 using EWGSOP1, whereas the sensitivity was of 16.7%, specificity of 84.7% and AUC of 0.58 using EWGSOP2. The MSRA-5 displayed a sensitivity of 3.2%, a specificity of 89.9% and an AUC of 0.41 using EWGSOP1, whereas sensitivity was of 0%, specificity of 91.1% and the AUC of 0.55 using EWGSOP2 criteria. The questionnaires showed a moderate agreement (Cohen's k = 0.53).

**Conclusions:**

In our sample of AD patients, a sizable number of sarcopenic individuals were misidentified by SARC-F-3 and MSRA-5, making those questionnaires unsuitable for sarcopenia screening. Considering that sarcopenia has a high prevalence in dementia and that its correct and timely identification is paramount for optimal management of patients, the development and validation of an ad-hoc sarcopenia screening tool for AD patients is highly desirable.

**Supplementary Information:**

The online version contains supplementary material available at 10.1186/s12877-022-03441-5.

## Background

Sarcopenia is a progressive and generalized skeletal muscle disorder, due to adverse muscle changes that occur over the course of life, characterized by loss of muscle mass, muscle strength and/or physical performance [1]. Sarcopenia is commonly observed among elderly but can also occur earlier in life [2]. The estimated prevalence of sarcopenia in community-dwelling older adults varies from 9.9 to 40.4%, depending on the definition adopted and has been show that individuals with neurocognitive disorder (NCD) may have a higher prevalence of sarcopenia features [3, 4].

Several definitions and diagnostic criteria for sarcopenia have been proposed over recent decades, with the first widely accepted being published in 2010 by the European Working Group on Sarcopenia in Older People (EWGSOP1) [5]. EWGSOP1 criteria differentiate presarcopenia, a condition characterized solely by loss of lean mass, sarcopenia, characterized by loss of lean mass and muscle strength or physical performance, and severe sarcopenia, when all three are present [3]. In 2018, a revised version of these criteria has been published (EWGSOP2) [2]. According to EWGSOP2, low muscle strength is considered the primary indicator of sarcopenia, while the presence of both low muscle strength and low muscle mass is necessary to confirm the diagnosis, and a concomitant loss of physical performance is indicative of severe sarcopenia [2]. In addition, EWGSOP2 introduced novel recommendations to improve early detection of sarcopenia in clinical practice, suggesting that muscle strength be assessed if patients report symptoms or signs of sarcopenia (feeling weak, slow walking speed, difficulty rising from a chair or weight loss/muscle wasting), preferably documented through the SARC-F (Strength, Assistance with walking, Rise from a chair, Climb stairs and Falls) questionnaire [2].

Recommendations on screening for sarcopenia are included also in the International Conference on Sarcopenia and Frailty Research guidelines, which suggested evaluating individuals aged 65 years or above annually or after the occurrence of major health events, by means of gait speed or SARC-F [6]. These indications mirror the growing awareness that a timely identification of sarcopenia is of the utmost importance, being the condition associated with negative health outcomes, including falls and fractures [7], mobility disorders and impairment in the activities of daily living [8], poor quality of life and mortality [9]. The SARC-F questionnaire and its abbreviated version (SARC-F-3) and the Mini Sarcopenia Risk Assessment Questionnaire and its abbreviated version (MSRA-5) are two widely used questionnaires for rapid sarcopenia screening in clinical practice [10, 11].

However, the number of studies investigating their accuracy in general population is still limited and, to the best of our knowledge, no studies have investigated their performance in patients with Alzheimer’s disease (AD), the most common type of dementia, where the prevalence of sarcopenia appears to be higher [4, 12]. Therefore, this study aims to fill this research gap by evaluating the performance of the SARC-F-3 and the MSRA-5 questionnaires in identifying sarcopenia in AD patients.

## Methods

### Study design

Single-center observational study.

### Study population

We enrolled 130 consecutive elderly adults who attended the Center for Neurodegenerative Diseases and the Aging Brain of the University of Bari Aldo Moro in Tricase between January 2019 and January 2020 and received a final diagnosis of mild or major NCD due to probable AD, based on the Diagnostic and Statistical Manual of Mental Disorders, fifth edition [13].

Fifty-two patients underwent lumbar puncture and showed evidence of AD pathophysiological process, thus presenting an increase level of diagnostic certainty according to the National Institute on Aging and Alzheimer's Association (NIA-AA) diagnostic guidelines [14].

Exclusion criteria were active treatment for cancer or cancer diagnosis in the past five years, severe knee or hip osteoarthritis limiting mobility, inflammatory diseases, stroke with upper and/or lower extremity involvement, Parkinson’s disease or other neurological disorders likely to interfere with physical function and major psychiatric illnesses. Only patients who were able to perform the handgrip test three times for each hand were included. More specifically, among 148 screened subjects, 3 were excluded for oncological pathologies, 8 for serious osteoarticular pathologies, 2 for inflammatory pathologies, 1 for recent stroke and 4 for inability to perform the handgrip test. Written informed consent was obtained from patients or from patient’s legal guardians if the patients were not able to provide it.

### Sarcopenia screening

SARC-F-3 and MSRA-5 were used to screen AD subjects regarding the risk of sarcopenia.

Participants' relatives/caregivers were present when the questionnaires were administered and allowed to help them with the responses, whenever necessary.

SARC-F is a five-item questionnaire, based on cardinal features of sarcopenia namely strength, assistance in walking, rise from a chair, climbing stairs and falls [15]. SARC-F-3 is the short form of SARC-F, comprising three of the five original items (strength, climbing stairs, and assistance in walking). Scores range from 0 to 6, and a SARC-F-3 score equal or higher than 2 is suggestive of increased risk of sarcopenia [10].

MSRA-7 comprises 7 items assessing age, physical activity level, number of hospitalizations during the preceding year, weight loss, number of meals per day, milk and dairy products, and protein consumption. The reduced version (MSRA-5) does not include questions on number of meals and milk and dairy products consumption. Score ranges from 0 to 60 (0, 5, 10 or 15 points for each item), and a score equal or lower than 45 is suggestive of increased risk of sarcopenia [11].

### Sarcopenia diagnosis

Sarcopenia was diagnosed according to EWGSOP1 and EWGSOP2 criteria, using their relative cut-off values for muscle mass and muscle strength [2, 5].

Skeletal muscle mass (SM) was estimated through a phase-sensitive single-frequency bioimpedance analyzer (BIA 101 Anniversary, Akern, Florence, Italy) [16]. Bioimpedance analysis was performed with the participant lying down on a testing table in a supine position and electrodes placed on the right hand and foot. Low muscle mass was defined as a Skeletal Muscle Index (SMI) computed as SM divided for height squared below or equal to 8.87 kg/m^2^ for males and to 6.42 kg/m^2^ for females for EWGSOP1, or as an appendicular skeletal muscle mass (ASM) below or equal to 20 kg for males and to 15 kg for females for EWGSOP2 [2, 5]. Muscle strength was evaluated through hand grip strength (HGS) using an electronic handheld dynamometer (DynX®, AKERN). HGS was assessed three times for each hand, alternating sides, and the best of the six grip strength measurements was registered. Low muscle strength was defined as an HGS below or equal to 30 kg for males and to 20 kg for females for EWGSOP1 and below or equal to 27 kg for males and to 16 kg for females for the EWGSOP2 criteria.

### Statistical analysis

Data were explored with descriptive statistics (mean ± SD or percentage). Sensitivity, specificity, positive likelihood ratio, negative likelihood ratio and the area under the receiver operating characteristic curve (AUC) were calculated for SARC-F-3 and MSRA-5 according EWGSOP1 and EWGSOP2 criteria. Cohen’s kappa (*k*) was used to assess the agreement between questionnaires. Statistical analyses were conducted using SPSS 19.0 (SPSS, Inc. Chicago, IL).

## Results

Demographic, clinic, and anthropometric data are reported in Table 1.

**Table 1:** Demographic, clinical characteristics and questionnaires score

	**Mean ± SD or Percentage**	**Range**
Male (%)	59 (45.4%)	
Age, *years*	70.71 ± 8.50	52–90
BMI, *kg/m*^*2*^	26.67 ± 4.14	18.12–39.26
MMSE	19.64 ± 6.26	2–30
Mild NCD (%) Major NCD (%)	43 (33.1%) 87 (66.9%)	
HGS, *kg*	19.31 ± 7.19	5–38.70
SMI, *kg/m*^*2*^	9.70 ± 1.78	5–14.88
ASM, *kg*	20.12 ± 4.41	11.60–34.30
EWGSOP1		
Low muscle mass, (%)	35 (26.9%)	
Low muscle strength, (%)	93 (71.5%)	
Sarcopenia (%),	31 (23.8%)	
EWGSOP2		
Low muscle mass (%)	18 (13.8%)	
Low muscle strength (%)	70 (53.8%)	
Sarcopenia (%)	6 (4.6%)	
SARC-F-3	0.72 ± 0.89	0–4
MSRA-5	53.81 ± 4.85	40–60

*BMI* Body Mass Index, *MMSE* Mini Mental State Examination, *NCD* Neurocognitive Disorder, *HGS* Hand Grip Strength, *SMI* Skeletal Muscle Index, *ASM* Appendicular Skeletal Muscle Mass, *EWGSOP1* European Working Group on Sarcopenia in Older People of 2010, *EWGSOP2* European Working Group on Sarcopenia in Older People of 2018, *SARC-F-3* Strength, Assistance with walking, Rise from a chair, Climb stairs and Falls questionnaire, 3-item version, *MSRA-5* Mini Sarcopenia Risk Assessment questionnaire, 5-item version

Forty-three patients (33.1%) met the diagnostic criteria for mild NCD due to probable AD and eighty-seven (66.9%) met the criteria for major NCD due to probable AD. Regarding MMSE score, 12 patients (9.2%) have a score between 2–10, 52 (40%) between 11–20 and 66 (50.8%) between 21–30.

All patients were community-dwelling at the time of evaluation. Sarcopenia was diagnosed in thirty-one (23.8%) and six individuals (4.6%) according to the EWGSOP1 and EWGSOP2 criteria, respectively. Differences in demographic, clinic, and anthropometric data with respect to presence of sarcopenia are reported in Supplementary Table 1 and Supplementary Table 2. A higher prevalence of sarcopenia was observed with EWGSOP1 (23,8%) compared to EWGSOP2 (4.7%) while prevalence of sarcopenia was similar between patients with minor and major NCD, regardless of the diagnostic criteria adopted. Sensitivity, specificity, positive likelihood ratio, negative likelihood ratio and the AUC for SARC-F-3 and MSRA-5 are reported in Table 2.

**Table 2:** Diagnostic performances of SARC-F-3 and MSRA-5

	**Sensitivity**	**Specificity**	**Positive Likelihood Ratio**	**Negative Likelihood Ratio**
**SARC-F-3**				
*Low muscle mass*				
EWGSOP1	8.6% (1.8–23.1)	82.1% (72.9–89.2)	0.48 (0.15–1.54)	1.11 (0.97–1.28)
EWGSOP2	27.8% (9.7–53.5)	86.6% (78.9–92.3)	2.07 (0.86–5.01)	0.8 (0.62–1.12)
*Low muscle strength*				
EWGSOP1	17.2% (10.2–26.4)	89.2% (74.6–96.9)	1.59 (0.57–4.45)	0.93 (0.80–1.07)
EWGSOP2	15.7% (8.1–26.4)	85.0% (73.4–92.9)	1.05 (0.47–2.36)	0.99 (0.86–1.15)
*Sarcopenia*				
EWGSOP1	9.7% (2.0–25.8)	82.8% (73.9–89.7)	0.56 (0.18–1.80)	1.09 (0.94–1.26)
EWGSOP2	16.7% (0.4–64.1)	84.7% (77.1–90.5)	1.09 (0.17–6.82)	0.98 (0.68–1.42)
**MSRA-5**				
*Low muscle mass*				
EWGSOP1	2.9% (0.1–14.9)	89.5% (81.5–94.8)	0.24 (0.04–2.04)	1.09 (0.99–1.19)
EWGSOP2	22.2% (6.4–47.6)	93.8% (87.6–97.5)	3.56 (1.16–10.93)	0.83 (0.65–1.07)
*Low muscle strength*				
EWGSOP1	7.5% (3.1–14.9)	89.2% (74.6–96.9)	0.70 (0.22–2.24)	1.04 (0.91–1.18)
EWGSOP2	2.9% (0.4–9.9)	85% (73.4–92.9)	0.19 (0.04–0.85)	1.14 (1.02–1.28)
*Sarcopenia*				
EWGSOP1	3.2% (0.1–16.7)	89.9% (82.2–95.1)	0.32 (0.04–2.40)	1.08 (0.98–1.18) 1.10 (1.04–1.16)
EWGSOP2	0% (0.0–45.9)	91.1% (84.7–95.5)	0	

*EWGSOP1* European Working Group on Sarcopenia in Older People of 2010, *EWGSOP2* European Working Group on Sarcopenia in Older People of 2018, *SARC-F-3* Strength, Assistance with walking, Rise from a chair, Climb stairs and Falls questionnaire, 3-item version, *MSRA-5* Mini Sarcopenia Risk Assessment questionnaire, 5-item version

Using EWGSOP1 criteria, the sensitivity and specificity of SARC-F-3 were 9.7% and 82.8%, whereas positive and negative likelihood ratio were 0.56 and 1.09. MSRA-5 showed a sensitivity and specificity of 3.2% and 89.9% with a positive likelihood ratio of 0.32, a negative likelihood ratio of 1.08. AUC was of 0.41 (95% CI: 0.31–0.52) for SARC-F-3 and of 0.41 (95% CI: 0.30–0.52) for MSRA-5. Using EWGSOP2 criteria, the sensitivity and specificity of SARC-F-3 were 16.7% and 84.7%, whereas positive and negative likelihood ratio were 1.09 and 0.98. MSRA-5 showed sensitivity and specificity of 0% and 91.1% with a positive likelihood ratio of 0 and negative likelihood ratio of 1.10. The AUC was 0.58 (95% CI: 0.35–0.81) for SARC-F-3 and 0.55 (95% CI: 0.34–0.76) for MSRA-5. Sensitivity and specificity analysis was also conducted separately in patients with mild and major NCD (data not shown) with analogous results. SARC-F-3 and MSRA-5 showed a moderate agreement (*k* = 0.53). More in detail 108 patients were identified as not at risk of sarcopenia by both SARC-F-3 and MSRA-5, 9 patients were identified as at risk of sarcopenia by both SARC-F-3 and MSRA-5, 11 patients were identified as at risk by SARC-F-3 and not at risk of sarcopenia by MSRA-5 while 2 patients were identified as at risk by MSRA-5 and not at risk by SARC-F-3.

## Discussion

This is the first study that assessed the performance of SARC-F-3 and MSRA-5 in screening patients with AD regarding the risk of sarcopenia. Overall, we found that both questionnaires showed low sensitivity, moderate-to-high specificity, and low AUC. Considering that these questionnaires have been proposed as tools for sarcopenia screening in clinical practice, the low sensitivity makes them unsuitable in patients with AD.

SARC-F-3 has been developed by Woo et al. based on the evidence that a combination of three out of the five questions of the original SARC-F showed the highest area under the curve and are able to predict all the adverse health outcomes (physical limitation, length of hospital stays, and mortality), among community-dwelling individuals [10].

Nevertheless, a subsequent study that compared the performance of SARC-F-3 and SARC-F in community-dwelling elderly (mean age 71.5 ± 5.8 y, sarcopenia diagnosed according to the Asian Working Group for Sarcopenia criteria) [17], showed that the SARC-F-3 displayed lower sensitivity (13.1%), specificity (97.8%) and AUC (0.676, 95% CI: 0.627–0.723) compared to SARC-F.

The excellent specificity and poor sensitivity for sarcopenia detection that the SARC-F-3 showed in our sample of AD patients is consistent with the results of previous studies.

However, up to now, no other studies have investigated its performance. On the other hand, several studies have assessed the performance of its extended version, among community-dwelling individuals. A recent metanalysis described that SARC-F has low-to-moderate sensitivity (28.9%-55.3%) and moderate-to-high specificity (68.9%-88.9%) and display slightly better accuracy than SARC-F-3 in identifying person at risk of sarcopenia. [18]

It has also been reported that the performance of SARC-F varies according to the specific diagnostic criteria adopted, with overall better performance observed with EWGSOP2 compared to EWGSOP1 [18–20]. Similarly, in our study the abbreviated version displayed greater sensitivity and specificity with EWGSOP2 (16.7% and 84.7%) than with EWGSOP1 criteria (9.7% and 82.8%). The differences in accuracy found using the two diagnostic criteria, as the different prevalence of sarcopenia, are likely attributable to the different cut-off points, more stringent for both muscle mass and muscle strength in EWGSOP2 compared to EWGSOP1. Nonetheless, further studies with a larger sample are needed to identify the factors associated to the differences in sarcopenia prevalence, as age, sex, cognitive performance, and functional ability.

The performance of SARC-F in patients with neurodegenerative diseases, so far, has been evaluated only in the study by da Luz et al. which administered the questionnaire to patients with Parkinson's disease (mean age 68.9 ± 6.5 y, sarcopenia diagnosed according to EWGSOP1 and EWGSOP2) and reported lower sensitivity (27,2%-23,1%) and specificity (66.6%-68.1%) [21], compared to general population. Taken together, these results seem to suggest that SARC-F is more suitable for screening regarding the risk of sarcopenia among hospitalized or nursing home elderly: indeed, community-dwelling elderly individuals are often able to perform most of the activities investigated by the questionnaire [22, 23]. The study by Cao et al. supports this view by showing that higher SARC-F scores are found in patients with older age, higher comorbidities and living in nursing homes [24].

Regarding MSRA-5, scientific literature is scant and generally carried out in community-dwelling elderly, with only few studies on hospitalized/institutionalized individuals. In community-dwelling individuals the MSRA-5 displayed a different sensitivity and specificity profile than SARC-F, presenting high sensitivity and moderate-to high specificity (80.4% and 64.4%, respectively in the original validation study) [11].

Studies directly comparing MSRA-5 and SARC-F confirmed these differences, reporting similar diagnostic accuracy but higher sensitivity and lower specificity of MSRA-5 than SARC-F [25–27]. However, a very recent study showed that, when MSRA-5 is used to identify sarcopenia among hospitalized individuals, the specificity is significantly lower (16.3%), although sensitivity remains high (90.9%) [28]. Our results on elderly non-hospitalized AD patients are in line with the above-mentioned study, showing a very low sensitivity and high specificity of MSRA-5, regardless the diagnostic criteria adopted. Collectively, these results seem to suggest that MSRA-5's performance is heavily influenced by clinical characteristics and living environment (community-dwelling vs hospitalized) of the population under examination. Therefore, the poor performance observed may be due to the fact that the characteristics investigated by the MSRA-5 are seldom presented by community-dwelling AD subjects. Finally, we found a moderate agreement between questionnaires (*k* = 0.53). Indeed, both questionnaires investigate complementary aspects of the same condition: SARC-F-3 evaluates subjective difficulties in physical performances, related to activity of daily living and muscle strength [29]; by contrast, MSRA-5 is based on objective indicators associated with increased risk of sarcopenia such as number of hospitalizations, activity level, age and nutrition [11].

In conclusion, the sensitivity and specificity profile found in our study indicates that a significant number of sarcopenic patients are misidentified by both SARC-F-3 and MSRA-5, thus not seem suitable as sarcopenia screening tools in AD.

Therefore, the development and validation of a reliable tool for sarcopenia detection in AD would be highly recommended, considering that individuals with cognitive decline are at increased risk of developing sarcopenia [30] and that this condition is potentially reversible through appropriate lifestyle changing interventions, mainly rehabilitation and proper nutrition [31, 32].

Several limitations of the present study should be acknowledged. First, physical performance was not evaluated, thus the prevalence of sarcopenia according to EWGSOP1 could be underestimated and the severity of sarcopenia (EGWSOP2) could not be assessed. Second, we use BIA to estimate muscle mass. Although BIA is mentioned among the techniques to estimate muscle mass in both the EWGSOP1 and EWGSOP2 criteria, the dual energy X-ray absorptiometry is the *gold standard* technique for quantifying muscle mass, due to its higher reproducibility and greater precision. Third, we did not administer the original version of the questionaries but only their abbreviated version. Finally, sample size is relatively small, although in line with that reported in several single-center studies, and or results need to be confirmed on a larger cohort of patients.

## Conclusions

SARC-F-3 and MSRA-5 do not seem suitable to screen AD patients regarding the risk of sarcopenia.

Considering that the prevalence of sarcopenia is high in AD, the development and validation of new screening tools that can be easily used in clinical practice is of paramount importance. In their absence, objective assessment of muscle mass, muscle strength, and physical performance is mandatory for correct identification of sarcopenia in AD patients.

## Supplementary Information

**Additional file 1:**

## Data Availability

The dataset supporting the conclusions of this article is available from the corresponding author upon reasonable request.

## Abbreviations

AD: Alzheimer’s Disease
ASM: Appendicular skeletal muscle mass
AUC: Area under the receiver operating characteristic curve
EWGSOP1: European Working Group on Sarcopenia in Older People of 2010
EWGSOP2: European Working Group on Sarcopenia in Older People of 2018
HGS: Hand grip strength
MSRA: Mini Sarcopenia Risk Assessment
MSRA-5: Mini Sarcopenia Risk Assessment – 5 items
NCD: Neurocognitive disorder
SARC-F: Strength, Assistance with walking, Rise from a chair, Climb stairs and Falls
SARC-F-3: Strength, Assistance with walking, Rise from a chair, Climb stairs and Falls – 3 items
SM: Skeletal muscle mass
SMI: Skeletal Muscle Index (SMI)
