# Supplementary material for: Sarcopenia screening in elderly with Alzheimer’s disease: performances of the SARC-F-3 and MSRA-5 questionnaires
Source: BMC Geriatr. 2022 Sep 17;22:761. doi: 10.1186/s12877-022-03441-5 (PMC9482268; doi:10.1186/s12877-022-03441-5)
Supplement: Supplementary file 1 — Additional file 1: [file 12877_2022_3441_MOESM1_ESM.docx]

***Supplementary Material***

**Sarcopenia screening in elderly with Alzheimer’s disease: performances of the SARC-F-3 and MSRA-5 questionnaires.**

Giulia Bramato, Roberta Barone, Maria Rosaria Barulli, Chiara Zecca, Rosanna Tortelli, Marco Filardi, Giancarlo Logroscino.

**Statistical analyses**

Differences in demographic, clinic, and anthropometric data with respect to presence of sarcopenia according to EWGSOP1 and EWGSOP2 criteria were analyzed by means of chi-squared and independent sample *t*-test (Supplementary Table 1 and Supplementary Table 2, respectively). Analyses were conducted with IBM SPSS Statistics 19 software (SPSS, Inc. Chicago, Ill); p value < 0.05 was considered statistically significant.

**Supplementary Table 1**

Demographic, clinical characteristics and questionnaires score according to sarcopenia diagnosed with EWGSOP1.

|  | **Sarcopenia**  (*n* = 31) | **No Sarcopenia**  (*n* = 99) | T_(3,142)_ | p-value |
| --- | --- | --- | --- | --- |
| *Demographic and clinical data* |  |  |  |  |
| Male/Female | 29/2 | 30/69 | 38.10 | < 0.0001 |
| Age, *y* | 68.65 ± 9.67 | 71.44 ± 8.04 | -1.61 | *ns* |
| BMI | 25.82 ± 3.46 | 27.01 ± 4.35 | -1.40 | *ns* |
| MMSE | 19.26 ± 6.90 | 19.79 ± 6.06 | -0.41 | *ns* |
| Mild NCD, (%)  Major NCD, (%) | 12 (27.9%)  19 (21.8%) | 31 (72.1%)  68 (78.2%) | 0.58 | *ns* |
|  |  |  |  |  |
| HGS, *kg*^a^ | 19.44 ± 6.44 | 19.27 ± 7.44 | 0.11 | *ns* |
| SMM^a^ | 27.19 ± 5.30 | 25.10 ± 7.03 | 1.52 | *ns* |
| ASM^a^ | 21.51 ± 3.56 | 19.68 ± 4.58 | 2.04 | < 0.05 |
| SARC-F^a^ | 0.48 ± 0.68 | 0.80 ± 0.94 | -1.73 | *ns* |
| MSRA-5^a^ | 55 ± 4.08 | 53.43 ± 5.03 | 1.58 | *ns* |

**Supplementary Table 2**

Demographic, clinical characteristics and questionnaires score according to sarcopenia diagnosed with EWGSOP2.

|  | **Sarcopenia**  (*n* = 6) | **No Sarcopenia**  (*n* = 124) | T_(3,142)_ | p-value |
| --- | --- | --- | --- | --- |
| *Demographic and clinical data* |  |  |  |  |
| Male/Female | 1/5 | 58/66 | 2.09 | *ns* |
| Age, *y* | 80.83 ± 7.78 | 70.29 ± 8.26 | 3.06 | < 0.005 |
| BMI | 24.48 ± 3.50 | 26.84 ± 4.18 | -1.36 | *ns* |
| MMSE | 19.50 ± 6.28 | 19.67 ± 6.27 | -0.06 | *ns* |
| Mild NCD, (%)  Major NCD, (%) | 2 (4.7%)  4 (4.6%) | 83 (95.4%)  41 (95.3%) | 0 | *ns* |
|  |  |  |  |  |
| HGS, *kg*^a^ | 14.02 ± 4.63 | 19.56 ± 7.20 | -1.86 | *ns* |
| SMM^a^ | 17.60 ± 4.18 | 25.98 ± 6.56 | -3.09 | < 0.005 |
| ASM^a^ | 14.08 ± 1.94 | 20.41 ± 4.29 | -3.59 | < 0.0005 |
| SARC-F^a^ | 1 ± 1.10 | 0.71 ± 0.88 | 0.78 | *ns* |
| MSRA-5^a^ | 53.33 ± 4.08 | 53.83 ± 4.90 | -0.24 | *ns* |
